# Clinical feasibility of endovascular recanalization with intravascular ultrasound-guided wiring for chronic total occlusion of below-the-knee arteries

**DOI:** 10.1186/s42155-023-00399-2

**Published:** 2023-10-19

**Authors:** Naoki Hayakawa, Satoshi Kodera, Hiromi Miwa, Shinya Ichihara, Satoshi Hirano, Masataka Arakawa, Yasunori Inoguchi, Shunichi Kushida

**Affiliations:** 1 Department of Cardiovascular Medicine, Asahi General Hospital, I-1326 Asahi, Chiba, 289-2511 Japan; 2 Department of Cardiovascular Medicine, The University of Tokyo Hospital, Tokyo, Japan

**Keywords:** Chronic total occlusion, Endovascular therapy, Intravascular ultrasound, Below-the knee, Scoring system

## Abstract

**Background:**

Revascularization with endovascular therapy (EVT) for complex below-the-knee (BTK) chronic total occlusion (CTO) remains a challenging problem. The Japanese-BTK (J-BTK) CTO score is reported as an indicator of the difficulty of BTK CTO, with the guidewire (GW) passage success rate decreasing as the grade increases. We previously reported an effective GW crossing method for the intravascular ultrasound (IVUS)-guided parallel wiring of complex BTK CTO. In this study, we investigated the feasibility of EVT using IVUS-guided wiring for BTK CTO.

**Materials and methods:**

This single center, retrospective study analyzed 65 consecutive BTK CTO vessels in which IVUS-guided wiring was attempted after the failure of a conventional antegrade wiring approach from November 2020 to November 2022. The primary endpoint was the clinical success of the target CTO vessel. The secondary endpoints were the GW success rate per grade based on the J-BTK CTO score, number of GW used for CTO crossing, fluoroscopy time, and complications.

**Results:**

Target vessels were the anterior tibial artery (66.2% of cases), peroneal artery (9.2%), and posterior tibial artery (24.6%). Blunt type CTO entry was performed in 55.4% of cases, calcification of entry was observed in 24.6% of cases, the mean occlusion length was 228.2 ± 93.7 mm, mean reference vessel diameter was 2.1 ± 0.71 mm, and outflow was absent in 38.5% of cases. J-BTK CTO scores of 0/1 (grade A), 2/3 (grade B), 4/5 (grade C), and 6 (grade D) were seen in 18.5%, 43.1%, 36.3%, and 1.5% of cases, respectively. The clinical success rate was 95.4%. The GW success rate by J-BTK CTO grade was as follows: grade A (100%), B (100%), C (91.7%), and D (0%). The mean number of GW used was 3.4 ± 1.4, the mean fluoroscopy time was 72.3 ± 32.5 min, and complications occurred in 7.7% of cases.

**Conclusion:**

This study showed a very high clinical success rate despite the difficulty of BTK CTO. IVUS-guided EVT might be a feasible strategy for complex BTK CTO.

## Background

Chronic limb-threatening ischemia (CLTI) requires revascularization by bypass surgery or endovascular therapy (EVT). Although there has been much debate and research on whether bypass surgery or EVT is the first choice for revascularization [1, 2], EVT is minimally invasive and has established itself as a useful modality for CLTI patients with a variety of comorbidities [3]. However, EVT for below-the-knee (BTK) artery chronic total occlusion (CTO) is one of the most challenging lesions to revascularize in CLTI patients [4]. Although the success of EVT for these types of lesions has been improved by the introduction of various retrograde approaches such as distal puncture (DP) or a trans-collateral approach (TCA), there are some cases where retrograde approaches are difficult to perform for lesions with poor distal target vessels [5]. For these types of lesions, bypass surgery is also difficult because of the presence of poor run-off vessels.

Tan et al. identified five variables: “no outflow of the target vessel”, “CTO length > 200 mm”, “reference vessel diameter (RVD) < 2.0 mm”, “calcification at the proximal entry point”, and “blunt type at entry point” as predictors of guidewire (GW) passage difficulty in BTK CTO and reported them as the Japanese-BTK (J-BTK) CTO score [6]. According to this scoring system, score 0/1 (grade A) and score 2/3 (grade B) have relatively high success rates of 97.3% and 76.8%, respectively, whereas score 4/5 (grade C) and score 6 (grade D) have much lower success rates of 19.3% and 0.0%, respectively. It is hoped that the introduction of novel approaches to these high-grade lesions will improve the success rate of these procedures.

We reported an extreme antegrade GW crossing using AnteOwl WR (AnteOwl) (Terumo, Tokyo, Japan) intravascular ultrasound (IVUS)-guided parallel wiring to a BTK artery as an EXCAVATOR technique to treat complex BTK CTO [7]. This new IVUS was developed for coronary artery CTO interventions [8]. Compared with conventional IVUS, this method had improved lesion crossability, durability, and image quality, and the asymmetrical structure of the IVUS transducer and IVUS on the wire makes it easier to locate the true lumen, making IVUS-guided wiring easy to perform, even for very small occluded vessels such as the BTK CTO. However, the success rate, complexity, and complications of our IVUS-guided wiring method for BTK CTO have not been studied, and whether this technique is feasible remains unclear. Therefore, in the present study, we investigated the clinical feasibility of EVT with IVUS-guided wiring for BTK CTO.

## Methods

### Study population and design

**Fig. 1:**
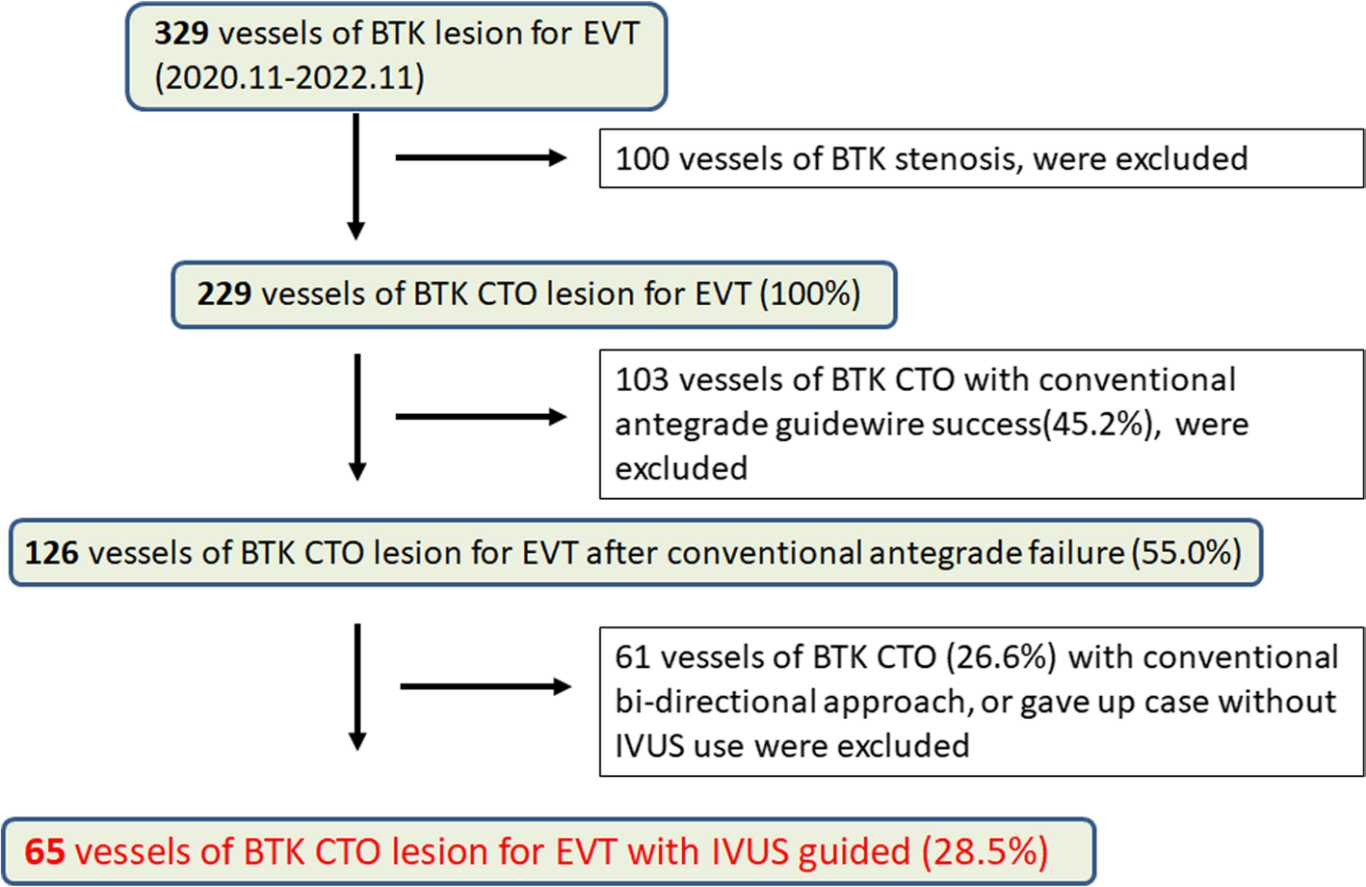
Study flow chart. BTK, below-the-knee; EVT, endovascular therapy; CTO, chronic total occlusion

The study was a single center, retrospective analysis. Between November 2020 and November 2022, 329 consecutive vessels of isolated BTK lesions of atherosclerotic disease were treated by EVT at Asahi General Hospital. “Isolated BTK lesion” indicates the CTO of BTK alone, and continuous or connected lesions of the femoropopliteal (FP) artery were excluded. Therefore, this study did not include continuous occlusion from the popliteal artery to the BTK arteries. However, cases in which BTK CTO and other FP lesions were observed and treated simultaneously were included. One hundred vessels of BTK stenosis were excluded, 103 vessels of BTK CTO with conventional antegrade GW success were excluded, and 61 vessels of BTK CTO with a conventional bidirectional approach, or cases where IVUS was not used were excluded. We retrospectively analyzed 65 consecutive vessels of BTK CTO for EVT with IVUS-guided wiring (Fig. 1). The selection of IVUS-guided wiring was decided based on each operator’s decision. The procedure was performed by three specialists from the Japanese Association of Cardiovascular Intervention and Therapeutics at our institute. The study protocol was approved by the local ethics committee of Asahi General Hospital, and the study was performed in accordance with the Declaration of Helsinki. The requirement for informed consent was waived because of the retrospective study design, in which existing medical records were used. Alternatively, patients could opt out of the study. Relevant information regarding the study is available to the public in accordance with the Ethical Guidelines for Medical and Health Research Involving Human Subjects.

### Procedural protocol

**Fig. 2:**
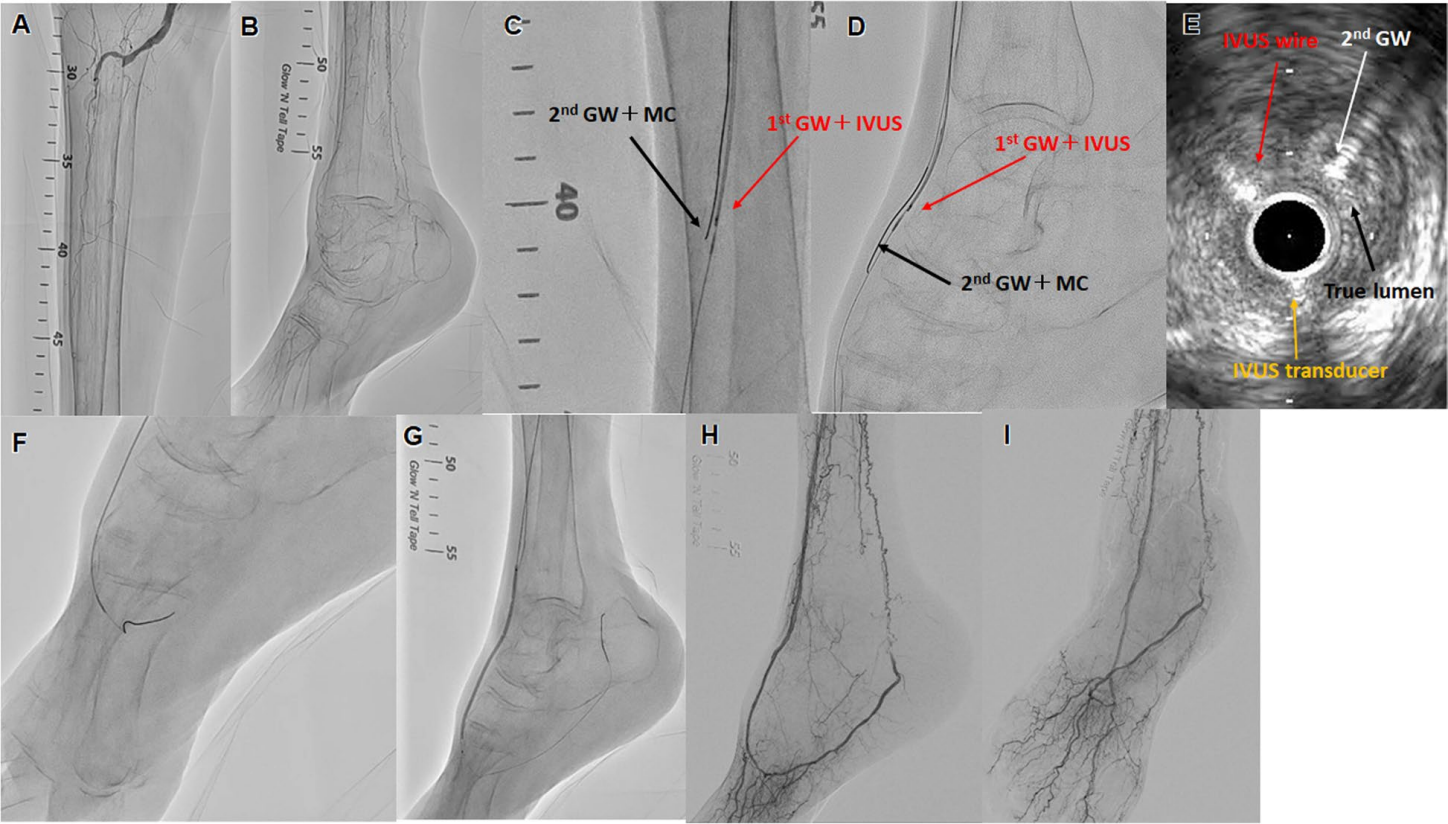
Successful revascularization with intravascular ultrasound (IVUS)-guided parallel wiring for complex below-the-knee (BTK) chronic total occlusion (CTO). **A**, **B** Control angiography shows total occlusion of both tibial arteries and very poor inframalleolar arteries. The target vessel was the right anterior tibial artery (ATA) to dorsalis pedis artery (DPA). **C**, **D** IVUS was placed on the first guidewire (GW) and the second GW and micro-catheter (MC) were advanced into the intraplaque of the ATA to DPA CTO lesion. Repeated IVUS-guided parallel wiring was performed to advance the true lumen of the DPA from the ATA. Red arrow: first GW with IVUS. Black arrow: second GW with MC. **E** IVUS findings of the CTO lesion in the DPA. White arrow: the second GW is in the true lumen. Black arrow: true lumen. Red arrow: IVUS on the guidewire. Yellow arrow: IVUS transducer. **F** The second GW was advanced into the distal true lumen. **G** IVUS measurement of the optimal size balloon dilation was performed. **H**, **I** Final angiography shows sufficient dilation, good antegrade blood flow, and small branches

Two antithrombotic drugs were used at least 24 h before the procedure was performed. Aspirin, clopidogrel, or prasugrel were mainly used. If there was a history of allergy or bleeding events after using those drugs, cilostazol was used as an alternative antiplatelet agent. Anticoagulants were used in cases where atrial fibrillation or other anticoagulants were needed. After the insertion of a guiding sheath from the ipsilateral or contralateral femoral artery, 0.014-inch GWs were used with a 2.6 to 2.8 Fr micro-catheter (MC). First, we performed conventional antegrade wiring and used IVUS for cases of difficult GW passage. After advancing the IVUS on the antegrade first GW to confirm a true or false lumen, the second GW and MC were brought into the CTO lesion for IVUS-guided parallel wiring. We defined the EXCAVATOR technique as a method of performing this step repeatedly using an antegrade approach [7] (Fig. 2. Bidirectional IVUS-guided wiring with antegrade IVUS and retrograde GW was also used in some cases. In some cases, IVUS-guided wiring was performed antegrade but failed to pass the lesion; thus, retrograde wiring was performed while observing the antegrade IVUS. The retrograde approach combined with the antegrade IVUS-guided wiring used DP or TCA, but no IVUS was performed retrogradely. The decision to combine IVUS-guided wiring and a bidirectional approach was made by the operator on a case-by-case basis. All IVUS were AnteOwl. After the successful passage of the GW, the vessel diameter was evaluated by IVUS and angiography, and the balloon dilation of optimal size was performed. Drug-coated balloons, stents, and atherectomy devices were not used in this study because they were not commercially available for BTK lesions in Japan during the study period. Therefore, even for cases of flow-limiting dissection, additional balloon dilation was performed.

### Study endpoints and definitions

The primary endpoint of this study was clinical success defined as < 50% residual stenosis without angiographic flow limitation. The secondary endpoints were 1) the GW success rate per grade based on J-BTK CTO score, 2) number of GWs used for CTO crossing, 3) fluoroscopy time, and 4) complications. The J-BTK CTO score consists of five components: a) proximal stump, b) calcification at the proximal entry point, c) RVD, d) CTO length, and e) outflow of the target vessel, as previously reported [6]. The score was determined using a six-point scale, with a) to d) being one point each and only e) being two points. Scores 0/1 were assigned as grade A, scores 2/3 were assigned as grade B, scores 4/5 were assigned as grade C, and a score of 6 was assigned as grade D. Calcification severity was evaluated by the Peripheral Arterial Calcium Scoring System grade [9]. FP and infrapopliteal (IP) lesion grading was assessed by the Global Limb Anatomical Staging System (GLASS) classification [10]. Inframalleolar (IM) lesion grading was assessed by GLASS classification and Kawarada’s classification [11]. Procedures and measurements of clinical events were performed at our institute by at least three specialists from the Japanese Association of Cardiovascular Intervention and Therapeutics.

### Statistical analysis

All statistical analyses were performed using JMP version 13.0 (SAS Institute, Cary, NC, USA). Data are presented as the mean ± standard deviation for continuous variables and as a percentage for categorical variables, unless otherwise indicated. In all analyses, *p* < 0.05 indicated statistical significance.

## Results

### Baseline characteristics

The clinical characteristics of patients are summarized in Table 1. The mean age was 81.1 ± 9 years and 40.0% were male. The prevalences of diabetes mellitus and hemodialysis were 73.8% and 7.7%, respectively. With regard to target CTO lesions, the anterior tibial artery (ATA) was observed in 27.7% of cases, ATA to dorsalis pedis artery (DPA) in 38.5% of cases, peroneal artery in 9.2% of cases, posterior tibial artery (PTA) in 18.5% of cases, and PTA to plantar artery (PLA) in 6.2% of cases.

**Table 1:** Baseline characteristics of patients

n	65
Age, years	81.05 ± 7.94
Male	26 (40%)
CLTI	65 (100%)
Ambulatory	21 (32.3%)
HT	61 (93.8)
DL	59 (90.8%)
DM	48 (73.8%)
CKD	21 (32.3%)
HD	5 (7.7%)
Wifi grade	
1	9 (13.8%)
2	15 (23.1%)
3	24 (36.9%)
4	18 (27.7%)
De novo	40 (61.5%)
ATA	18 (27.7%)
ATA-DPA	25 (38.5%)
PA	6 (9.2%)
PTA	12 (18.5%)
PTA-PLA	4 (6.2%)
FP lesion treated	22 (34.4%)
Entry blunt	36 (55.4%)
Calcification at entry point	16 (24.6%)
PACCS 3/4	9 (13.8%)
Lesion length	270.1 ± 78.2
CTO length	228.2 ± 93.7
RVD	2.1 ± 0.71
Proximal RVD < 2.0	36 (55.4%)
Outflow absent	25 (38.5%)
J-BTK CTO	
0	4 (6.2%)
1	8 (12.3%)
2	13 (20.0%)
3	15 (23.1%)
4	17 (26.2%)
5	7 (10.1%)
6	1 (1.5%)
GLASS FP grade	
0	38 (58.5%)
1	20 (30.8%)
2	2 (3.1%)
3	2 (3.1%)
4	3 (4.6%)
GLASS IP grade	
2	2 (3.1%)
3	8 (12.3%)
4	55 (84.6%)
GLASS stage	
II	6 (9.2%)
III	59 (90.8%)
Pedal arch	
P0	8 (12.3%)
P1	42 (64.6%)
P2	15 (23.1%)
Kawarada classification	
1	7 (10.8%)
2A	14 (21.5%)
2B	27 (41.5%)
3	17 (26.2%)

*CLTI* chronic limb threatening ischemia, *HT* hypertension, *DL* dyslipidemia, *DM* diabetes mellitus, *CKD* chronic kidney disease, *HD* hemodialysis, *ATA* anterior tibial artery, *DPA* dorsalis pedis artery, *PA* peroneal artery, *PTA* posterior tibial artery, *PLA* plantar artery, *FP* femoropopliteal, *PACSS* Peripheral Arterial Calcium Scoring System, *CTO* chronic total occlusion, *RVD* reference vessel diameter, *J-BTK* Japanese-below-the-knee, *GLASS* Global Limb Anatomical Staging System

De novo lesions were present in 61.5% of cases. Blunt-type CTO entry occurred in 55.4% of cases, calcification at the proximal entry point was present in 24.6% of cases, the mean RVD was 2.1 ± 0.71 mm, the mean lesion length was 270.1 ± 78.2 mm, the mean CTO length was 228.2 ± 93.7 mm, and outflow was absent in 38.5% of cases. GLASS IP class 4 was observed in 84.6% of cases and GLASS stage III was observed in 90.8% of cases. GLASS IM/pedal descriptor P1 was observed 64.6% of cases and P2 was observed in 23.1% of cases. Rates of Kawarada classification were as follows: type 2A (21.5% of cases), 2B (41.5%), and 3 (26.2%). The grades of J-BTK CTO were as follows: A (18.5% of cases), B (43.1%), C (36.3%), and D (1.5%). BTK and FP lesions were treated simultaneously in 34.4% of cases.

### Outcome measures

**Fig. 3:**
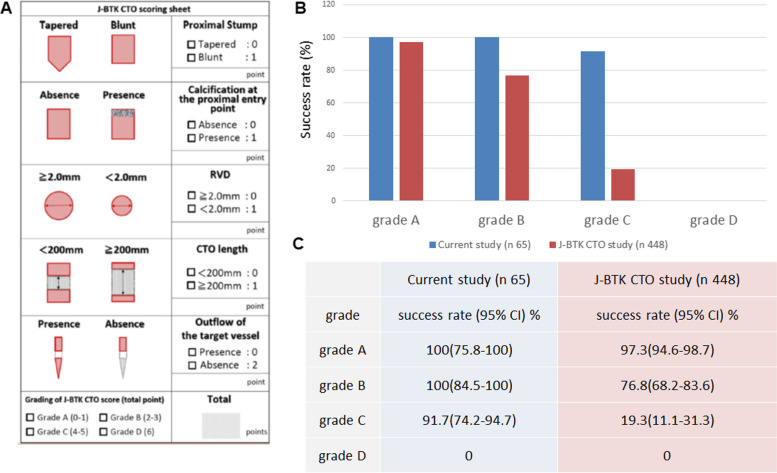
Japanese below-the-knee chronic total occlusion (J-BTK CTO) scoring sheet and comparison of clinical results between the J-BTK CTO study and current study. **A** Reproduced with permission from Tan et al. J Vasc Surg 74(2):506–513.e2. 10.1016/j.jvs.2021.01.059. **B** Comparison of clinical results between the J-BTK CTO study and current study. Blue bar graph shows the results of this study. Red bar graph shows the results of the J-BTK CTO study. **C** Comparison between the current study and previously reported success rate by J-BTK CTO score grade using 95% confidence intervals

The procedural outcomes are summarized in Table 2. Clinical success of the primary outcome was achieved in 95.4% of cases and the GW success rate was 95.4%. The success rate of the antegrade approach alone was 63.1%. DP was required in 15.4% of cases. All cases requiring DP were those that could not pass GW with antegrade IVUS-guided wiring alone. DP was performed using an angiography-guided approach with a 21G metal needle, and a 2.6Fr micro catheter was inserted. The sheath was not inserted from retrograde in any case. The mean number of GWs used for CTO crossing was 3.4 ± 1.4, the mean maximum GW tip load was 16.2 ± 14.7 g, and the mean fluoroscopic time was 72.3 ± 32.5 min. Procedural complications were observed in 7.7% of cases, branch perforation in 4.6% of cases, vessel oozing perforation in 1.5% of cases, and access site bleeding in 1.5% of cases. Perioperative mortality was 0%. The GW success rates for each J-BTK CTO grade compared with previous reports are shown in Fig. 3. The clinical and GW success rates with 95% confidence intervals (CI) in the current study by J-BTK CTO grade are as follows: grade A (100% [95% CI: 75.8–100]), B (100% [95% CI: 84.5–100]), C (91.7% [95% CI: 74.2–94.7]), and D (0%) (Fig. 3B, C).

**Table 2:** Procedural characteristics

n	65
Clinical success	62 (95.4%)
GW success	62 (95.4%)
Antegrade only	41 (63.1%)
Bidirectional	22 (33.8%)
Distal puncture	10 (15.4%)
Trans pedal approach	11 (16.9%)
EXCAVATOR technique	49 (79.0%)
0.014-inch guidewire	65 (100%)
AnteOwl WR IVUS use	65 (100%)
CTO GW maximum tip load	16.2 ± 14.7
Method of GW crossing	
Drilling or penetration	58 (89.2%)
Knuckle wiring	4 (6.2%)
GW number for CTO	3.4 ± 1.4
Fluoroscopy time	72.3 ± 32.5
Procedural complication	5 (7.7%)
Branch perforation	3 (4.6%)
Vessel oozing perforation	1 (1.5%)
Access site bleeding	1 (1.5%)
Procedural death	0 (0.0%)

*GW* guidewire, *EXCAVATOR* extreme antegrade guidewire crossing by AnteOwl WR intravascular ultrasound-guided parallel wiring to a BTK artery, *IVUS* intravascular ultrasound, *CTO* chronic total occlusion

## Discussion

The present study demonstrated the clinical feasibility of EVT with IVUS-guided wiring for BTK CTO. The clinical and GW success rates were very high at 95.4%. Because this study dealt with cases in which GW passage was not successful using an antegrade method with a conventional angiography-guided approach, an even higher procedural success rate for the total number of BTK CTOs is to be expected. Furthermore, the GLASS and Kawarada classifications of the lesions suggest that many of the lesions were very complex in this study.

Previous studies of FP lesions reported that IVUS-guided EVT was useful in improving clinical outcomes [12, 13]. In the BTK area, previous studies reported the usefulness of assessing the optimal vessel diameter [14, 15], but no studies have examined the use of IVUS-guided wiring for the passage of CTO lesions. To the best of our knowledge, this is the first clinical study of the use of IVUS-guided wiring for BTK CTO.

A previous report showed a relatively high rate of antegrade GW crossing failure in BTK CTO lesions [16]. In general, if an antegrade GW fails to advance into the true distal lumen, a retrograde approach might be considered including DP or TCA. Other clinical reports described the efficacy of retrograde approaches for BTK CTO [17, 18]. However, in some cases, such as those with lesions with poor distal target vessels, a retrograde approach is very difficult to perform. Furthermore, the higher the J-BTK CTO score, the lower the procedural success rate [6]. Factors that contribute to the failure of these procedures include the morphology and length of the occlusion, calcification, small vessel diameter, and poor outflow vessel,thus, IVUS-guided wiring may be a possible solution to these problems. Indeed, we observed a marked improvement in GW success rates for grades B and C of the J-BTK CTO score compared with previous reports [6]. The results by J-BTK CTO score were compared between the current study and previously reported results with 95% CI. For grades B and C, the estimated lower limits of the current study were both higher than the upper limits of the previously reported [6] results, suggesting the present study’s method is more beneficial than the previously reported methods (Fig. 3).

As reported in our previous studies [7], AnteOwl IVUS was used for all cases in this study. This IVUS was originally developed for coronary CTO interventions. The profile of the transducer was 2.6Fr, and the length from the tip to the transducer was 8 mm. The IVUS catheter has a durable coating, double monorail lumen, and the transducer and IVUS guidewire have asymmetrical structures. The frequency of IVUS was 40 MHz. It is also very useful for complex peripheral vascular CTO lesions because of its small profile and excellent lesion crossability, high-resolution imaging, and asymmetric tip structure that allows navigation of the second GW within the CTO. We treated BTK CTO using other IVUS prior to the current study period, but experienced many cases of difficulty related to lesion passage or evaluation of the true lumen because of the poor image quality. The quality of the procedure is expected to improve dramatically with the use of AnteOwl IVUS.

In the current study, the tip load of GW for CTO crossing exceeded 16 g. This is a GW with a rather stiff tip, and the risk of vessel perforation is high by tactile sensation alone. However, it is assumed that the second GW could be manipulated while grasping the correct position of the true lumen to be targeted by IVUS, and thus the lesion could be successfully passed through using such a stiff GW. The use of a stiff GW while confirming the lesion with IVUS suggests that even a lesion that could not be passed into the true lumen in an antegrade fashion with a standard GW could be successfully passed. There are also concerns about complications such as large vessel dissection or vessel perforation caused by the advancing IVUS into the subintima and the use of a stiff GW to target the true lumen. However, this study had no fatal complications and only minor complications, including bleeding in the side branches.

The success rate of the antegrade approach alone was 63.1%, with 36.9% of cases requiring a bidirectional approach in the current study. This does not represent a purely antegrade success rate, as some of these cases required a strictly antegrade approach alone, but others included cases where a combination of antegrade and retrograde approach would be simpler to cross the lesion. However, DP, a representative retrograde approach, was used in about 15% of the cases, despite the complexity of the lesion after the failure of conventional antegrade wiring. This is clearly attributable to the improved accuracy of antegrade wiring using the IVUS-guided method. Reduced DP and TCA rates may reduce the risk of injury to distal healthy vessels, and may also contribute to anastomotic preservation when transitioning to bypass surgery after EVT. In addition, the IVUS-guided approach is not only an antegrade procedure; when it is combined with a retrograde approach, it can lead to success in more complex cases, which is expected to significantly increase the total procedure success rate.

Another merit of IVUS-guided wiring is the possibility of increasing the distance of GW passage from the true lumen. Although no studies have compared the intima and subintima patency in BTK CTO, in general, patency is difficult to achieve with balloon dilation alone in the subintima passage because of immediate recoil. We experienced a case in which many side branches were found and good blood flow was observed after all BTK CTOs were passed through the true lumen (Fig. 2). Future studies on the contribution of this method to long-term patency and wound healing are warranted.

**Fig. 4:**
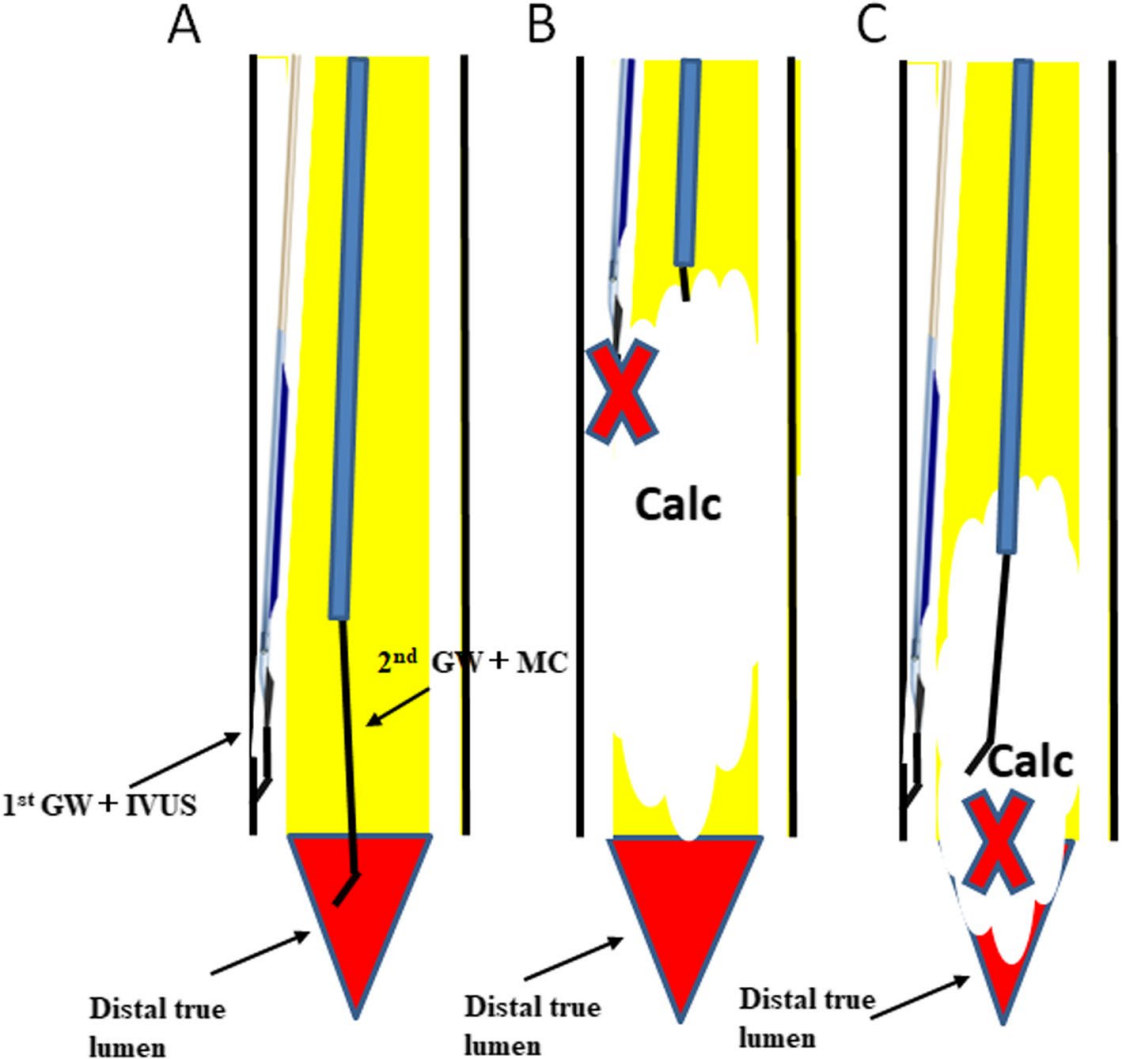
A comparison of the success and failure of intravascular (IVUS)-guided wiring for below-the-knee (BTK) chronic total occlusion (CTO). **A** Success rates are higher for lesions that can be brought distally to the IVUS catheter. **B**, **C** If IVUS cannot pass through due to calcification (**B**), or if IVUS can pass through but the distal true lumen is calcified (**C**), it is difficult to pass the lesion despite using IVUS-guided wiring

Although this technique is highly feasible for BTK CTO lesions, the difficulty of the procedure is much greater than that of FP CTO, and it tends to require more procedure time and number of GW. Indeed, although this study included 34.4% of cases in which FP lesions had to be treated simultaneously, the fluoroscopy time exceeded 70 min and it took more than 3 GWs to pass the CTO. Furthermore, this IVUS-guided technique also has limitations depending on the anatomy of the CTO lesion. Success rates were higher for lesions that were brought distally to the IVUS catheter (Fig. 4A). However, if the IVUS cannot advance because of calcification (Fig. 4B), or if the IVUS can pass through but the distal true lumen is occluded by calcification (Fig. 4C), the procedure is difficult. In this study, there was a 0% success rate for cases with a grade D J-BTK CTO score. These cases are so-called no-option CLTI, and it may be better to consider alternatives such as percutaneous deep vein arterialization [19]. In the present study, cases such as those in the study flow (Fig. 1), in which the use of IVUS was considered difficult from the beginning, were not used and were therefore excluded. Cases in which even a MC could not be advanced because of severe calcification, or cases in which calcification filled the periphery, were considered as cases where the use of IVUS could have been abandoned at the start of treatment. Because these cases were excluded, the success rate of the procedure may have been overestimated, and future studies of IVUS-guided wiring in all-comers may be necessary.

The most important aspects of BTK intervention are wound healing and avoidance of major amputations. Successful revascularization contributes to these clinical outcomes, as previous studies have shown [20, 21]. However, this study examined the passage of GW to CTO and did not collect clinical data on subsequent wound healing or avoidance of major amputation. Future analysis should also investigate whether these factors improve clinical outcomes.

This study had several limitations. It was a retrospective, nonrandomized study with a small sample size; therefore, the evidence level was not high. All clinical events were evaluated on-site and there was no independent clinical events committee. The application of IVUS-guided wiring was selected based on each operator’s decision without following any pre-established protocol. There may have been selection bias. This procedure is difficult, and therefore, there will be a learning curve for the operator. Although each operator in this study was experienced in BTK EVT, individual operator proficiency might have influenced the clinical outcomes. In addition, this was a single-arm study of IVUS-guided wiring for BTK CTO, and no comparative study with other methods has been conducted. Therefore, comparative analyses with other methods or conventional wiring strategy are needed to examine the effectiveness and safety of this technique. In the future, a well-designed, large-scale prospective study will be required for equivalent evaluation.

## Conclusions

Our study showed that EVT with IVUS-guided wiring for BTK CTO might be a feasible strategy for the treatment of BTK CTO lesions. This approach could be a new GW crossing strategy for BTK CTO lesions following DP and TCA.

## Data Availability

The datasets used and/or analyzed during the current study are available from the corresponding author upon reasonable request.

## Abbreviations

CLTI: Chronic limb-threatening ischemia
EVT: Endovascular therapy
BTK: Below-the-knee
CTO: Chronic total occlusion
DP: Distal puncture
TCA: Trans-collaterals approach
RVD: Reference vessel diameter
GW: Guidewire
J-BTK: Japanese-below-the-knee
AnteOwl: AnteOwl WR
IVUS: Intravascular ultrasound
EXCAVATOR: Extreme antegrade guidewire crossing by AnteOwl WR intravascular ultrasound-guided parallel wiring to a BTK artery
MC: Micro-catheter
FP: Femoropopliteal
IP: Infrapopliteal
GLASS: Global Limb Anatomical Staging System
IM: Inframalleolar
ATA: Anterior tibial artery
DPA: Dorsalis pedis artery
PTA: Posterior tibial artery
PLA: Plantar artery
CI: Confidence interval

## References

[1] Farber A, Menard MT, Conte MS, Kaufman JA, Powell RJ, Choudhry NK (2022). Surgery or endovascular therapy for chronic limb-threatening ischemia. N Engl J Med.

[2] Bradbury AW, Moakes CA, Popplewell M, Meecham L, Bate GR, Kelly L (2023). A vein bypass first versus a best endovascular treatment first revascularisation strategy for patients with chronic limb threatening ischaemia who required an infra-popliteal, with or without an additional more proximal infra-inguinal revascularisation procedure to restore limb perfusion (BASIL-2): an open-label, randomised, multicentre, phase 3 trial. Lancet.

[3] Iida O, Takahara M, Soga Y, Kodama A, Terashi H, Azuma N (2017). Three-year outcomes of surgical versus endovascular revascularization for critical limb ischemia: the SPINACH study (surgical reconstruction versus peripheral intervention in patients with critical limb ischemia). Circ Cardiovasc Interv.

[4] Lyden SP (2009). Techniques and outcomes for endovascular treatment in the tibial arteries. J Vasc Surg.

[5] Schmidt A, Bakker OJ, Bausback Y, Scheinert D (2017). The tibiopedal retrograde vascular access for challenging popliteal and below-the-knee chronic total occlusions: literature review and description of the technique. J Cardiovasc Surg (Torino).

[6] Tan M, Ueshima D, Urasawa K, Hayakawa N, Dannoura Y, Itoh T (2021). Prediction of successful guidewire crossing of below-the-knee chronic total occlusions using a Japanese scoring system. J Vasc Surg.

[7] Hayakawa N, Kodera S, Hirano S, Arakawa M, Inoguchi Y, Kanda J (2022). An AnteOwl WR intravascular ultrasound-guided parallel wiring technique for chronic total occlusion of below-the-knee arteries. CVIR Endovasc.

[8] Okamura A, Iwakura K, Iwamoto M, Nagai H, Sumiyoshi A, Tanaka K (2020). Tip detection method using the new IVUS facilitates the 3-dimensional wiring technique for CTO intervention. Cardiovasc Interv.

[9] Rocha-Singh KJ, Zeller T, Jaff MR (2014). Peripheral arterial calcification: prevalence, mechanism, detection, and clinical implications. Catheter Cardiovasc Interv.

[10] Conte MS, Bradbury AW, Kolh P, White JV, White JV, Dick F, Fitridge R (2019). Global Vascular Guidelines on the Management of Chronic Limb-Threatening Ischemia. Eur J Vasc Endovasc Surg.

[11] Kawarada O, Fujihara M, Higashimori A, Yokoi Y, Honda Y, Fitzgerald PJ (2012). Predictors of adverse clinical outcomes after successful infrapopliteal intervention. Catheter Cardiovasc Interv.

[12] Tsubakimoto Y, Isodono K, Fujimoto T, Kirii Y, Shiraga A, Kasahara T (2021). IVUS-guided wiring improves the clinical outcomes of angioplasty for long femoropopliteal CTO compared with the conventional intraluminal approach. J Atheroscler Thromb.

[13] Allan RB, Puckridge PJ, Spark JI, Delaney CL (2022). The impact of intravascular ultrasound on femoropopliteal artery endovascular interventions: A randomized controlled trial. JACC Cardiovasc Interv.

[14] Fujihara M, Yazu Y, Takahara M (2020). Intravascular ultrasound-guided interventions for below-the-knee disease in patients with chronic limb-threatening ischemia. J Endovasc Ther.

[15] Soga Y, Takahara M, Ito N, Katsuki T, Imada K, Hiramori S (2021). Clinical impact of intravascular ultrasound-guided balloon angioplasty in patients with chronic limb threatening ischemia for isolated infrapopliteal lesion. Catheter Cardiovasc Interv.

[16] Kokkinidis DG, Strobel A, Jawaid O, Haider MN, Alvandi B, Singh GD (2020). Development and validation of a predictive score for anterograde crossing of infrapopliteal chronic total occlusions: (the infrapop-CTO score). Catheter Cardiovasc Interv.

[17] Rogers RK, Dattilo PB, Garcia JA, Tsai T, Casserly IP (2011). Retrograde approach to recanalization of complex tibial disease. Catheter Cardiovasc Interv.

[18] Bazan HA, Le L, Donovan M, Sidhom T, Smith TA, Sternbergh WC (2014). Retrograde pedal access for patients with critical limb ischemia. J Vasc Surg.

[19] Shishehbor MH, Powell RJ, Montero-Baker MF, Dua A, Martínez-Trabal JL, Bunte MC (2023). Transcatheter arterialization of deep veins in chronic limb-threatening ischemia. N Engl J Med.

[20] Nakama T, Watanabe N, Haraguchi T, Sakamoto H, Kamoi D, Tsubakimoto Y (2017). Clinical outcomes of pedal artery angioplasty for patients with ischemic wounds: results from the multicenter RENDEZVOUS registry. JACC Cardiovasc Interv.

[21] Kobayashi N, Hirano K, Yamawaki M, Araki M, Takimura H, Sakamoto Y (2017). Clinical effects of single or double tibial artery revascularization in critical limb ischemia patients with tissue loss. J Vasc Surg.

